# Community Perceptions on Integrating Animal Vaccination and Health Education by Veterinary and Public Health Workers in the Prevention of Brucellosis among Pastoral Communities of South Western Uganda

**DOI:** 10.1371/journal.pone.0132206

**Published:** 2015-07-28

**Authors:** Catherine Kansiime, Lynn M. Atuyambe, Benon B. Asiimwe, Anthony Mugisha, Samuel Mugisha, Victor Guma, Innocent B. Rwego, Elizeus Rutebemberwa

**Affiliations:** 1 Department of Health Policy, Planning and Management, Makerere University School of Public Health, College of Health Sciences, Kampala, Uganda; 2 Department of Community Health and Behavioral Sciences, Makerere University School of Public Health, College of Health Sciences, Kampala, Uganda; 3 Department of Medical Microbiology, College of Health Sciences, Makerere University, Kampala, Uganda; 4 Department of Veterinary Medicine, College of Veterinary Medicine, Animal Resources and Bio-security, Makerere University, Kampala, Uganda; 5 Department of Biological Sciences, College of Natural Sciences, Makerere University, Kampala, Uganda; 6 Department of Mental Health and Community Psychology, Makerere University College of Humanities and Social Sciences, Kampala, Uganda; 7 Ecosystem Health Initiative, College of Veterinary Medicine, University of Minnesota, St. Paul, Minnesota, United States of America; Centers for Disease Control and Prevention, UNITED STATES

## Abstract

**Background:**

Brucellosis is a zoonotic disease of veterinary, public health, and economic significance in most developing countries, yet there are few studies that show integrated human and veterinary health care intervention focusing on integration at both activity and actors levels. The aim of our study, therefore, was to explore community perceptions on integration of animal vaccination and health education by veterinary and public health workers in the management of brucellosis in Uganda.

**Methods:**

This study used a qualitative design where six Focus Group Discussions (FGDs) that were homogenous in nature were conducted, two from each sub-county, one with the local leaders, and another with pastoralists and farmers. Five Key Informant Interviews (KIIs) with two public health workers and three veterinary extension workers from three sub-counties in Kiruhura district, Uganda were conducted. All FGDs were conducted in the local language and tape recorded with consent from the participants. KIIs were in English and later transcribed and analyzed using latent content data analysis method.

**Results:**

All the groups mentioned that they lacked awareness on brucellosis commonly known as Brucella and its vaccination in animals. Respondents perceived improvement in human resources in terms of training and recruiting more health personnel, facilitation of the necessary activities such as sensitization of the communities about brucellosis, and provision of vaccines and diagnostic tests as very important in the integration process in the communities. The FGD participants also believed that community participation was crucial for sustainability and ownership of the integration process.

**Conclusions:**

The respondents reported limited knowledge of brucellosis and its vaccination in animals. The community members believed that mass animal vaccination in combination with health education about the disease is important and possible if it involves government and all other stakeholders such as wildlife authorities, community members, local to national political leaders, as well as the technical personnel from veterinary, medical and public health sectors since it affects both humans and animals.

## Introduction

Brucellosis is a zoonosis of veterinary, public health and economic significance in most developing countries [1]. It affects human health, as well as the health and reproductive performance of livestock, thus reducing their productivity, leads to abortions and weak offspring and is a major impediment for trade and export [2]. Studies on animal brucellosis in Uganda have reported a herd prevalence of 55.6% and animal prevalence of 15.8% in the pastoral dairy system in Mbarara district [3], while higher figures of up to 100% at herd level and 30% at animal level have been reported in the Central district of Nakasongola [4]. Recently, a cross sectional study of the disease in two sub counties of Kazo and Buremba in Ibanda district, Western Uganda, found a prevalence of 38.4% and 26% in cows and goats respectively [5]. Human brucellosis has been reported to be prevalent in both rural and urban settings [6,7] and a more recent study revealed that 12.6% of informally marketed milk in urban Kampala was contaminated with *B*. *abortus* at purchase; and the annual human incidence rate in Kampala was estimated to be 5.8 per 10,000 people [8].

The explosive human population growth and environmental changes have resulted in increased numbers of human-animal interactions which presents a potential zoonotic threat [9] that may cause spillover of infections from wildlife to cattle in areas around national parks. Brucellosis is also prevalent among the Ugandan wildlife populations [10], and other studies have reported prevalence of brucellosis in buffaloes at 10% in Egypt and 5.05% in Pakistan [11] with evidence of spill over to outdoor reared domestic pigs and cattle.

With increasing human-animal interface, integration of services is critical to encourage working together of both public health and veterinary professionals. Integration is at the heart of systems theory and, therefore, central to organizational design and performance [12]. Despite the fact that systems are comprised of separate hierarchical structures with interconnected components; these components are supposed to play complementary roles in order to accomplish their joint tasks [13]. However, the division, decentralization, and specialization found in many organizations usually interfere with efficiency and quality goals [14]. Therefore, the fulfillment of system aims necessitates co-operation and collaboration among and between the various parts of the system. In this sense, integration is the “glue” that bonds the entity together, thus enabling it to achieve common goals and optimal results [15].

There is no universal definition of integration, but various researchers have defined it differently. Gröne and Garcia-Barbero [16] sums up health care integration as the “bringing together of inputs, delivery, management and organization of services as a means of improving access, quality, user satisfaction and efficiency.” Brown & McCool [17] suggest that integration allows for greater efficiency and effectiveness, less duplication and waste, more flexible service provision, and better co-ordination and continuity. On the other hand, the Program for Appropriate Technology in Health [18] defines health services integration as the organization, management, and delivery of a continuum of preventive and curative health services.

While great successes have been achieved through vertical programming notably in childhood immunizations, HIV/AIDS and tuberculosis diagnosis and care in Tanzania, as well as integrated care for HIV/AIDS and maternal and child health in Kenya [18], it is recognized that integration of health services can derive greater overall impact from health resources and systems.

Traditionally, prevention of human brucellosis has mainly depended on control of the disease in domestic animals [19], with scholars arguing that these are the main reservoir of the disease [20]. A study in Greece [21] found that vaccination of animals against brucellosis combined with health education on preventive measures among agricultural and pastoral population led to a reduction in the incidence of human brucellosis. However, in areas bordering conservation areas, vaccination is complicated by the domestic animals—wildlife interaction since there are no vaccination efforts for the wildlife species [22]. In Uganda, *Brucella* S19 vaccine is used against animal brucellosis with emphasis on cattle as a response to a reported outbreak. The focus is mainly on *B*. *abortus*. Vaccination is a voluntary exercise, but government also provides the vaccine through Ministry of Agriculture, Animal Industry and Fisheries (MAAIF) and it is also done by District Local Government headed by the District Veterinary Officer during disease outbreak. There is no test and slaughter policy in place, due to the related costs of farmer compensation. Despite the significant role of vaccination in the prevention of brucellosis, there is a need to introduce other measures such as health education on the nature and mode of transmission of the disease. It is important to note that good knowledge of the disease is essential for the success of a control program as documented elsewhere [23]. In a study of non-sedentary pastoralist communities, Ward et al [24] recommended health education, especially on zoonoses, as one branch of collaboration between veterinary and public health services in such populations.

In communities where livelihoods depend on domestic animals and their products, such as the pastoral rangelands of South Western Uganda, human-animal interaction is intimate. There is therefore a need to greatly recognize that animal and human health are closely linked and that veterinary and public health sectors share the common goal of protecting, promoting and improving the health and wellbeing of human populations [25]. Closer co-operation between human and animal healthcare providers can also lead to financial savings in the two sectors [26]. In resource poor countries, control measures targeting zoonotic diseases at the human-domestic animal-wildlife interface could work provided that they are designed and adopted by the local population [27]. There are few studies of integrated human and veterinary health care intervention focusing on integration at both activity and actors levels. Those commonly described often deal with delivery of health care under special circumstances such as human disease outbreak necessitating both local and international funding [24]. The aim of our study therefore was to explore community perceptions on integration at two levels: the activity level which focused on animal vaccination and health education, and the actor level which involved veterinary and public health workers in the management of brucellosis among pastoralist communities living in close proximity with wildlife in Kiruhura district, South Western Uganda.

### Conceptual framework on Approach to Integrated Health Services PATH 2011[18]

This framework encompasses four levels of a country’s health structure—client-centered services at the community level, health operations planning at the organization level, health system coordination at the national level, and inter-sectoral initiatives across development sectors.

Client-centered services at the facility and community levels involves integrated programming that fit the needs of clients, including individuals and families, as well as the broader community. It may involve increased awareness of brucellosis in terms of symptoms, improved access to treatment services, drugs, preventive methods both in animals and humans.

Operational elements at the health organization levels, often requires changes in how services are delivered by ministries of health, nongovernmental or local organizations, and private-sector agencies. Existing or new health system inputs (such as resources, time, money, or expertise) may need to be allocated differently to support planning, management, staffing, interpersonal communication, or the measurement of integrated services.

Broader governance and capacity issues at the health system level such as; new levels of coordination or joint planning of the policies, processes, and infrastructure that make up a health system may be needed to deliver integrated services. Integration at this level often requires significant involvement and support from all stakeholders, including donors, ministries of health, politicians, advocacy groups, the private sector, and non-governmental organizations.

Inter-sectoral coordination such as cross-sector integration is when public health personnel jointly work with veterinary extension staff to provide health education on brucellosis during animal vaccination. Integration across sectors requires engagement and commitment at multiple levels of the organization as discussed above.

The strength of this framework is that it looks at integration of services in a wider perspective to include all sectors as well as client needs. However, it fails to describe challenges likely to be encountered by different sectors in integration of services. Awareness of likely challenges is an important issue for successful integration.

## Methods

### Study area and population

The study was conducted in the pastoralist communities of Nyabushozi County, Kiruhura district, South Western Uganda. Three sub-counties of Kanyaryeru to the West, Nyakashashara to the East, and Sanga to the North of Lake Mburo National Park (LMNP) were purposively selected. This was because of their close proximity to wildlife which poses a potential risk for zoonotic spill overs. The communities in this region derive their livelihoods from consumption and selling of cattle and their products but have recently started growing food crops at a subsistence level. The economic livelihoods in this area include: the farmers who are settled and solely grow crops (mostly the recent immigrants to the area); the agro-pastoralists who rear cattle as well as practice crop farming, and lastly the pure pastoralists/ semi-nomads who only rear cattle but have permanent shelters, only moving their animals in dry seasons in search of water and pasture. The majority of cattle keepers in this area, however, are agro-pastoralists. In this area, animals are mainly vaccinated against Foot and Mouth Disease, and treated against Trypanosomiasis, and Lumpy Skin Disease and this is only done during suspected disease outbreaks.

### Study design and sampling

This was a qualitative study that utilized two data collection methods namely: Focus Group Discussions (FGDs) and Key Informant Interviews (KIIs). Six FGDs of 10 participants each and five KIIs were conducted. These were purposively selected. The FGDs included local leaders (local council chairpersons, councilors, opinion leaders, religious leaders, as well as youth and women representatives), pastoralists and farmers. The KIIs comprised of veterinary and public health practitioners.

### Data Collection

Focus Group Discussions (FGDs) refer to a qualitative methodology where people with similar background or experiences gather to discuss a specific topic of interest with a researcher [28]. The researcher gains understanding of a particular issue from the perspective of the group participants [29]. This technique allows a small group of participants to discuss issues led by a moderator using a discussion guide [30]. On average, the number of participants in each FGD is recommended to be at least eight [31]. In the current study, 57 of the 60 expected FGD participants turned up.

The discussions were held at a venue, in each of the three sub-counties, identified by and convenient to the participants. Prior to data collection, study tools were pre-tested so as to help identify emerging themes. Highlighted below are the key topics identified and later explored during the FGDs and KIIs: i) community perception on integration of animal vaccination and health education on brucellosis (at activity level), ii) community perceptions on integration of the two services by veterinary and public health workers (at actor level), iii) how the integration can be conducted (at both levels), iv) challenges likely to be faced as well as recommendations to overcome them (at both levels).

The FGD guide was translated from the original English version into the local language (Runyankole) by Makerere University Institute of Languages. All FGDs were conducted in the local language and tape recorded with verbal consent from the participants. The moderator introduced the topic and aims of the study, and guided the discussions which lasted for one and a half hours until saturation where participants repeated the same information when they were asked the questions again. Recordings were later transcribed into English by the note taker and the principal investigator.

A total of five key informants were purposively selected on the basis of their professions and experience in order to provide an overview on the integration process. Two Clinical Officers (these are in-charge of Health Centre IIIs at sub-county level) and three Veterinary Officers (who are in-charge of sub-counties and responsible for veterinary extension services to the farmers) were interviewed to supplement the FGDs. The KIIs were conducted at the venue that was convenient to them (their place of work). Semi-structured interviews were conducted with Key Informant (KIs) because of their position or experience, and in-depth knowledge [32,33] on animal vaccination and health education on brucellosis. When respondents are well informed people, they provide an overall view of the community [34]. We also used KIIs to discover the subjective meanings and interpretations of peoples’ experiences. In this case, people’s responses are less influenced by the presence of their peers [35].

All KIIs were conducted in English and lasted an average of 45 minutes each. The first author (CK) conducted the interviews which were tape recorded with consent from the participants. Later the interviews were transcribed verbatim by the note taker and the first author. Tape recording was used in this study because it allows more time for eye contact between the moderator and the respondent during the interview [36] and the interviewer was able to get details and accuracy that would not be ascertained from field notes alone.

### Data management and analysis

The unit of analysis was the transcripts from the FGDs and the KIIs. Transcripts were first read through several times while making notes on them. All transcripts were cross-checked to ensure completeness of data and were kept under lock and key. Content analysis using both Latent and manifest analysis were used; Latent analysis technique refers to what the text talks about and involves in-depth interpretation of the underlying meanings of the text while manifest content analysis refers to the analysis of the obvious visible components [37,38]. Data were condensed without losing quality as described above.

All transcripts were read though and data were discussed by the authors who came up with codes. Responses with similar codes were re-categorized under a unifying sub-theme or theme. A matrix was created and individual matrices were discussed by the authors (CK and VG) until an agreement was reached. The categories were then interpreted for their descriptive meaning. Descriptive quotes representing key themes were identified by the authors and included in the final report writing.

### Ethical Statement

The study protocol was approved by Makerere University School of Public Health Higher Degrees, Research and Ethics Committee as well as Uganda National Council for Science and Technology. The study objective was explained to participants in their local language (Runyankole), written consent to participate in the study was obtained from all individuals before the discussions and interviews begun. Permission was also asked verbally from participants to have the sessions recorded and they were informed that the recordings were for the researchers only. For anonymity and confidentiality in the information provided, participants were assured that their names would not be used in analysis. Participants were informed that their participation was voluntary and they were free to withdraw anytime. Each focus group discussion was held within the village where the participants came from and at a time and place of their choice. The key informant interviews were conducted at a time and place convenient to each person.

## Results

This study set out to explore community perceptions on the integration of animal vaccination and health education by veterinary and public health workers in the management of brucellosis among pastoralists of Kanyaryeru, Sanga and Nyakasharara sub-counties in Kiruhura district, South Western Uganda.

### Socio-demographic characteristics of the participants

A total of 57 participants from three sub-counties of Nyakashahara, Sanga and Kanyaryeru were recruited into the Focus Group Discussions (FGDs) of which 51(89.5%) were males. The mean age of the participants was 46 years with a standard deviation of 10.13. There were five key informants (including two females).

The key themes that the research targeted were: i) Community perceptions on integration of animal vaccination and health education on brucellosis (the activity level), ii) community perceptions on integration of the two services by veterinary and public health workers (the actor level), iii) how to conduct the integration process (at both levels) and, iv) challenges likely to be faced as well as recommendations to overcome them at both levels (S1 File). These themes are discussed below:

### Community perceptions on integration of animal vaccination and health education on brucellosis (the activity level)

In the study area, brucellosis is commonly known as *Brucella*. From the discussions held with the community members, lack of awareness on brucellosis such as signs, symptoms, prevention, mode of transmission and vaccination in animals was highlighted and perceived as a limitation to the integration process. Five out of the six FGDs believed the integration process will work if the community is provided with adequate information on brucellosis.

“*We have never heard about animal vaccination against Brucella*. *We would like to know more about it and about the signs and symptoms because when our people and cattle are suffering from Brucella*, *we cannot tell unless the doctor or veterinary doctor tests and tells us*. *We therefore want to know more about Brucella in humans and animals*, *the cause*, *symptoms and how we can prevent it*. *This is important for integration because you cannot integrate what the people do not know”* (FGD with farmers and pastoralist, Kanyaryeru sub-county).

Respondents expressed their concerns about the free movements of wild animals in the communities. They believed that integration of animal vaccination and health education alone would not be effective in the prevention of brucellosis unless the wild animals are restricted. This is illustrated below:

“*First*, *government and park management should work towards restricting the wild animals from our communities by fencing them in the park because without doing that*, *the disease will remain in the communities”* (KII veterinary officers, Sanga and Kanyaryeru sub-counties).

“*Before you think of reducing Brucella in humans you must first reduce it in wild animals and livestock because they are the source of the disease*. *Only when this is done should we proceed with health education and vaccination of our animals”* (FGD local leaders, farmers and pastoralists, Nyakashashara and Kanyayeru sub-county).

Four out of five FGDs and two key informants believed that animal vaccination against brucellosis is more important than health education as illustrated below:

"*When you tell us to stop drinking unboiled milk and other practices*, *it is difficult to stop*. *For instance*, *I cannot burn all the cow dung to kill all bacteria nor will I fail to help my cow to deliver because you say I should not*. *The important thing to do is to vaccinate our animals against Brucella and later teach us about the disease since animals are the source of the disease*. *Otherwise*, *teaching us about the disease will not help us if our animals remain sick”* (FGD local leader, Nyakashashara sub-county).

*“The main focus should be on mass vaccination of animals against brucellosis because they are the main source of disease*. *When this is done*, *it will contribute to the reduction of the disease in humans and then health education comes in as an additional informative action to further reduce brucellosis in the communities”* (KII veterinary officer, Kanyaryeru sub-county).

### Community perceptions on integration of the two services by veterinary and public health workers (the actor level)

During the discussions and interviews, participants were given an opportunity to express their opinions regarding the integration of the two services by the veterinary and health workers. Participants believed that veterinary and health personnel could work together but they emphasized the need to also train local people especially in disseminating information about brucellosis in churches, at parties and on radios:

"*The health providers and veterinary doctors will need to train at least two people in every parish about Brucella in humans and animals*. *These could spread the information across the villages*, *in churches*, *and at parties*. *Information should also be broadcasted on the radios especially Radio Five*, *Radio West and Radio Uganda; these are the most listened to in this locality”* (FGD local leaders, Kanyaryeru sub-county).

All the FGDs and key informants mentioned shortage of veterinary officers as a limitation in the integration process. The participants believed that there is need for facilitation in terms of more health providers and veterinarians if the integration process is to succeed.

“*I think the veterinary officers should be facilitated by training and recruiting more of them because there is only one veterinary doctor in each sub-county*. *This would help them work with public health workers to provide joint services to the communities*. *I think if government increased their numbers and extended them to the communities they would be able to teach and treat animals”* (FGD farmers and pastoralists, Sanga sub-county and KII veterinary officer, Kanyaryeru sub-county).

Focus group participants and KII respondents were engaged and allowed to discuss their opinions and perceptions on how to integrate the two services by the veterinary and public health workers in the management of brucellosis. Joint provision of the services by both veterinary and public health workers was mentioned as good for the management of brucellosis in the community. It was suggested that this could be achieved through joint programming of activities by veterinary and health providers.

"*I think if the two services are integrated in the providers’ different programs*, *they will be able to work together*, *otherwise this would be the biggest challenges*. *For example*, *the public health workers could incorporate health education of Brucella in their outreach programs and also work with veterinary officers jointly during animal vaccination to teach people about Brucella in humans”* (KII veterinary, Kanyaryeru sub-county).

Gender issues were raised as important in the integration at activity level. Participants mentioned gender roles of women in handling animal products and care seeking for children as important opportunities to teach them about brucellosis while for men, animal vaccination would be the best way to capture and educate them about the disease at vaccination sites.

"*Integrated services are important for instance*, *when people go for outreach programs like immunization it would be good if veterinary officers would come along and teach the women who come for antenatal or bring children for immunization about boiling milk*, *water*, *and proper cooking of animal-derived foods*. *The women are involved in cooking*, *handling of most of the animal products and hygiene issues*. *If veterinary officers could organize activities at the sub-county such as testing and vaccinating animals and government intervenes by providing more veterinary officers*, *the integration process would work”* (FGD local leaders, Sanga and Kanyaryeru sub-counties).

"*Provide continuous free animal vaccination against brucellosis so that more men can be captured during vaccination and given health education about the disease since most men do not attend outreach programs and this would be one of the ways to involve them in the integration process”* (KII veterinary officer, Sanga sub-county).

All KII respondents believed that government and stakeholder involvement in the integration at actor level was important since veterinary and public health workers would not manage on their own. They highlighted the role of government in sensitization, training and improving human resources.

"*Involve Ministry of Health*, *district*, *sub-county*, *local council members and the private sector in the planning*, *management and implementing of these services in the communities”* (KII veterinary officer, Sanga sub-county).

*“Government should be involved in the vaccination process by providing free Brucella vaccines like it does during outbreaks for foot and mouth disease or provide the vaccine at subsidized prices because it is very expensive and pastoralists cannot afford it”* (KII veterinary officer, Kanyaryeru and Nyakashashara sub-counties).

Four FGDs and all Key Informants believed that animal vaccination and health education about brucellosis is not enough unless it is accompanied by infrastructural development. They highlighted the need for laboratory facilities for rapid diagnosis and treatment of humans; and vaccination or test and slaughter for their livestock in the integration process as illustrated below:

*“Integration of the two services to be successful needs establishment of equipped laboratories centered at the sub-counties to facilitate testing for both the livestock and the humans*. *This enables us prevent the disease and increases access to diagnosis in humans and treatment (vaccination in animals because Brucella is rampart in these areas as seen from the numerous animal abortions*, *infertility and weak calves”* (FGD local leaders, Kanyaryeru sub-county).

*“Veterinarians can closely work with health providers to provide the two services but this should be well planned with more diagnostic laboratories for both animals and humans and equipping these facilities is essential since the disease is often misdiagnosed”* (KIs senior clinical officer, and Veterinary officer Sanga and Kanyaryeru sub-counties).

### Challenges likely to be faced during the integration process as well as recommendations for successful integration

During the discussions and interviews, a number of challenges likely to be encountered during the integration process at both the actor and activity levels were believed to be; workload, human resource shortage and facilitation problems.

"*The problems the veterinary and health personnel may face include overload of work*, *for instance*, *the public health workers have other programs they conduct during their outreaches*, *and veterinary officers also have their different schedules to conduct in the communities and this may not fit into the other's schedule unless they are supported by government to incorporate animal vaccination and health education of Brucella into their programs and then*, *the two professionals agree on when*, *and where to conduct jointly these two services”* (FGD leaders, farmers and pastoralists, Kanyaryeru sub-county).

Inadequate human resources, especially fewer veterinary personnel, were believed to be a big hindrance to vaccination and unless this is addressed, the integration at actor level may not be possible.

"*In this sub-county*, *we have about 500*,*000 cattle but only one veterinary doctor who is also the District Veterinary Officer*. *You do not expect him to handle all the animals in the communities*. *It is important therefore that government increases the number of veterinary officers at sub-county level if the integration is to work”* (FGD local leaders, farmers and pastoralists, Nyakashashara sub-county).

*“More manpower especially the veterinary officers and more training for both providers would be needed to facilitate the integration program”* (KII Senior Clinical Officer, Nyakashashara sub-county).

Four out of five key informant respondents emphasized the need to facilitate the veterinary and health personnel in form of allowance and transport as important for the integration process.

***“*** *Transport costs are one major problem since our areas have a poor road network and the people are sparsely populated*. *For the integration process to work*, *facilitation will be needed in form of allowances and transport”* (KII Senior Clinical Officer, Nyakashashara and Sanga sub-counties).

All the Key Informants mentioned that brucellosis vaccines in animals were very expensive and not readily available making it unaffordable by most of the pastoralists with the exception of a few who request for it.

"*We carry out animal vaccination against brucellosis only at the request of the farmers*. *This service is private and not free*, *and only about five people request for it in a month*. *The challenges are failure to control brucellosis because the vaccine is expensive and usually unavailable*. *There are no vaccine programs for brucellosis yet there is a high population of susceptible animals in this sub county*: *about 500*,*000 cattle*, *40*,*000 goats and 10*,*000 sheep plus wildlife”* (KII veterinary officer, Sanga sub-county).

In regard to human brucellosis, the participants acknowledged that brucellosis services such as health education and treatment are more readily available as compared to information and vaccination against the disease in animals. For instance, five FGDS were not aware of the symptoms, transmission and availability of animal vaccination against brucellosis. However, all the participants mentioned unavailability of both animal and human diagnostics tests at the community level.

## Discussion

Community and provider perceptions on the integration of health education and animal vaccination against brucellosis by both public health workers and veterinarians were explored in our study. These focused on integration of animal vaccination and health education on brucellosis (the activity level), integration of the two services by veterinary and public health workers (the actor level), as well as how to conduct the integration process, and challenges likely to be faced. It was noted that integration of animal vaccination and health education would be feasible if government and other stakeholders were involved in facilitation of the programs. Key areas of improvement mentioned included; restricting wildlife movements in the communities, facilitating the necessary activities such as sensitization of the communities about brucellosis, provision of brucellosis vaccines and diagnostic tests as well as collaborations between all stakeholders to improve human resources.

All the Focus Group participants were not aware of vaccination against brucellosis, neither had they administered, nor done it for their animals. Five out six of the groups in our study believed that lack of knowledge about brucellosis was a hindering factor to integration; as a result, the community needs to first understand the importance of the integration before it is implemented. An earlier study done in the same area [39] reported moderate and low community knowledge on human and animal brucellosis respectively. Elsewhere, a study in Saudi Arabia found that awareness of the disease and its routes of transmission among livestock keepers may, on occasions, be poor [40]. It is important to note that good knowledge of the disease is essential to the success of a control program [23] as well as mass vaccination of livestock [2]. Ward et al, [24] recommended health education, especially on zoonoses as one branch of collaboration between veterinary and public health services. Therefore, efforts should be made to equip the veterinary and public health workers and the general community with adequate knowledge of brucellosis on; symptoms, improved access to treatment services, drugs, preventive methods both in animals and humans in order for integrated programming to fit the needs of clients, including individuals and families, as well as the broader community.

All the participants as well as the key informants believed that integration of animal vaccination would be effective if free movement of wildlife in the community was restricted to reduce the spread of brucellosis. This was attributed to close proximity and increased interaction with livestock. Increased interaction at the wildlife-human—livestock interface could be because of prolonged droughts that force wild animals to move to the communities as well as pastoralists moving their animals to the park in search of water and pasture. A study done by Lake Mburo National Park Authority in 2009 reported that farmers, mostly from Sanga, Kanyaryeru, and Nyakashashara sub-counties, moved over 15,000 cattle to the park that competed with wild animals for food and water and increased the risk for the transmission of animal diseases [41]. Although, wildlife brucellosis represents a potential zoonotic threat [9] which may cause spillover of infections to cattle in areas around national parks, no vaccine has proven to be safe and to provide significant degree of protection in wildlife species [22]. Therefore, there is need for community and park authorities to work together to allow temporary and safe access to Lake Mburo National Park during dry seasons, because it is not just wildlife transgressing boundaries but also the humans pushing into the wildlife space.

Despite the significant role of vaccination in the prevention of brucellosis, there is a need to combine vaccination with other measures such as health education on the nature and mode of transmission of the disease. This could be done through joint collaborations on policies, processes, and infrastructure which may be needed to deliver integrated services. Integration at this level, requires existing or new health system inputs (such as resources, time, money, or expertise) to be allocated differently to support planning, management, staffing, interpersonal communication, or the measurement of integrated services. And within the broader health system, new levels of coordination or joint planning of the policies, processes, and infrastructure that make up a health system may be needed in the delivery of the integrated services [18]. This often requires significant involvement and support from all stakeholders, including donors, ministries of health, politicians, advocacy groups, the private sector, and non-governmental organizations to effectively integrate these services and manage brucellosis in the communities.

Respondents in our study believed that veterinary and health personnel could work together but they emphasized the need to also train local people especially in disseminating information about brucellosis. Training of local community members such as village health teams to assist in mobilization and dissemination of information was highlighted as important for community participation and sustainability of the integration process. Elsewhere [2], it has been noted that community participation and joint collaboration between public health workers and veterinary officers is crucial for the acceptability and sustainability of effective control of brucellosis.

Respondents and participants believed that better general education and information on brucellosis and animal vaccination would help in the integration process and this could best be disseminated using radio broadcasting, in church announcement, parties and training village teams. A study done in nomadic pastoralists communities in Chad [42] noted that communities preferred that information on future vaccination campaigns be directed to their representatives and the complementary mode of information transmission mentioned was radio broadcasting. Therefore, animal vaccinations should be accompanied by continuous health education on the disease especially to those directly involved in the animal and food processing sector in order to guarantee sustainability of the campaigns [43]. This could be done through the media, advertisement and social gathering.

Integration of animal vaccination and health education by the veterinary and public health workers is believed to be important in the management of brucellosis in communities if it is jointly provided. However, the key informants and group participants believed that adequate funding and joint planning by the two professions in terms of time and resources would be key to the success of the effort. A study done in Greece [44] among agricultural and pastoral populations found that vaccination of animals against brucellosis combined with health education led to a reduction in the incidence of human brucellosis from 13.2 per 1,000 population in 1997 to 0.7 per 1,000 population in 2002, a drop of 12.5% in five years.

Joint programming of animal vaccination and health education is believed to be important for successful integration in communities. For example, three respondents mentioned that outreach programs such as child immunization days, antenatal care and HIV counseling and testing would be useful to capture more people especially women because they are the ones mainly involved in handling animal products at household level. A study done in Southern Sudan [24] found that increased coverage for childhood vaccination under the Expanded Programs for Immunization was achieved when livestock owners brought their cattle for vaccination against rinderpest at the same time and in the same location. Additionally, other authors [45] have proposed joint veterinary and health services in order to reach pastoralists in remote zones, so as to reduce costs and increase acceptance. Therefore, there is need to encourage joint service provision for both animals and humans by both veterinary and public health workers for successful provision of services in pastoral communities of Uganda.

Major challenges to the integration process, according to the respondents and group participants in our study, would be the lack of diagnostic facilities and tests for both humans and animals and inadequate veterinary staffs. They reported having only one government veterinary officer at each sub-county yet the animal population is large. All the key informants highlighted the high costs of brucellosis vaccine and its unavailability as a challenge in animal vaccination. In most developing countries, control of brucellosis in cattle is not straight forward as mass vaccination and stumping out programs are not feasible or sustainable in terms of cost [46]. Significant joint efforts, both in terms of human and financial resources as well as time, therefore needs to be allocated differently to support planning, management, staffing, interpersonal communication, or the measurement of integrated services in the management of brucellosis. This should be accompanied by an increase in the number of human resource especially veterinary officers as well as providing affordable, simple and rapid screening tests for brucellosis that can be used outside the established laboratory.

In our study, we modified the “Approach to Integrated Health Services” adapted from PATH 2011 [18] to discuss our findings. The strength of this framework is that it looks at integration of services in a wider perspective to include all sectors as well as client needs. However, it fails to describe challenges likely to be encountered by different sectors in integration of services. Awareness of likely challenges is an important issue for successful integration. In our study, the community members were able to articulate likely challenges as seen in the discussion section above. There were no major differences in opinion between the farmers and the local leaders group, as they were able to agree on a common approach.

### Methodological considerations

The strength of our study is that it was conducted among pastoralists who are exposed to zoonotic diseases such as brucellosis, and their experiences contributed to their perceptions on the integration of animal vaccination and health education in the management of the disease. The use of triangulation of methods (FGDs, KIIs and a previous knowledge study in the same area [39]) brought about a more accurate assessment of the phenomena studied by testing the consistency in the diversity of perspectives on integration of services both at the activity and actors level as recommended elsewhere [35]. Possible limitations were; we did not specifically conduct KII with policy makers to enable us capture the logistical barriers to integration of activities across sectors from the policy perspective and there were also few key informants especially the veterinarians to give a wider overview of the communities on the integration process. This was due to few veterinary officers in the area (one government veterinary extension worker per sub-county) and unavailability of health providers during the study period.

For further studies, we recommend that future epidemiological surveys in this setting should the assess role of wildlife in the epidemiology of brucellosis and other zoonotic diseases.

## Conclusion

The respondents reported limited knowledge of brucellosis and its vaccination in animals. The community members believed that mass animal vaccination in combination with health education about the disease is important and possible if it involves government and all other stakeholders such as wildlife authorities, community members, local to national political leaders, as well as the technical personnel from veterinary, medical and public health sectors since it affects both humans and animals.

## Supporting Information

S1 File Focus Group Discussion and Key Informant interview guide. To explore the perceptions and acceptability of integrating animal vaccination and health education by veterinary and health workers in the prevention of brucellosis among pastoral communities adjacent to Lake Mburo National Park.
(DOCX)
