# Supplementary material for: Community Perceptions on Integrating Animal Vaccination and Health Education by Veterinary and Public Health Workers in the Prevention of Brucellosis among Pastoral Communities of South Western Uganda
Source: PLoS One. 2015 Jul 28;10(7):e0132206. doi: 10.1371/journal.pone.0132206 (PMC4517904; doi:10.1371/journal.pone.0132206)
Supplement: S1 File — To explore the perceptions and acceptability of integrating animal vaccination and health education by veterinary and health workers in the prevention of brucellosis among pastoral communities adjacent to Lake Mburo National Park. (DOCX) [file pone.0132206.s001.docx]

**Focus Group Discussion and Key Informant interview guide**

**To explore the perceptions and acceptability of integrating animal vaccination and health education by veterinary and health workers in the prevention of brucellosis among pastoral communities adjacent to Lake Mburo National Park.**

1. What does animal vaccination mean to you and what does it involve? (Probe about who does it, how often, why they do it and the challenges they face?).
2. What kind of information would you want included in health education on brucellosis? (Probe about the ways this information can be disseminated to reach all the people and the local people’s contribution in making it a success).
3. What are your opinions on the integration of animal vaccination and health education by the veterinary and health workers in the management of brucellosis? (Probe about their understanding and importance of health education, health and veterinary workers in jointly providing these services, how the integration may be conducted, when and where and the role of the community in the integration).
4. What challenges do you think would arise in the integration process of animal vaccination and health education by the veterinary and health providers in the management of brucellosis? (probe on recommendations to address the challenges mentioned).
